# Acidic pH promotes intervertebral disc degeneration: Acid-sensing ion channel -3 as a potential therapeutic target

**DOI:** 10.1038/srep37360

**Published:** 2016-11-17

**Authors:** Hamish T. J. Gilbert, Nathan Hodson, Pauline Baird, Stephen M. Richardson, Judith A. Hoyland

**Affiliations:** 1 Wellcome Trust Centre for Cell-Matrix Research, Division of Cell Matrix Biology and Regenerative Medicine, School of Biological Sciences, Faculty of Biology, Medicine and Health, University of Manchester, Michael Smith Building, Oxford Road, Manchester, M13 9PT UK; 2 Division of Cell Matrix Biology and Regenerative Medicine, School of Biological Sciences, Faculty of Biology, Medicine and Health, University of Manchester, Stopford Building, Oxford Road, Manchester, M13 9PL UK; 3 NIHR Manchester Musculoskeletal Biomedical Research Unit, Central Manchester NHS Foundation Trust, Manchester Academic Health Science Centre, Manchester, United Kingdom

## Abstract

The aetiology of intervertebral disc (IVD) degeneration remains poorly understood. Painful IVD degeneration is associated with an acidic intradiscal pH but the response of NP cells to this aberrant microenvironmental factor remains to be fully characterised. The aim here was to address the hypothesis that acidic pH, similar to that found in degenerate IVDs, leads to the altered cell/functional phenotype observed during IVD degeneration, and to investigate the involvement of acid-sensing ion channel (ASIC) -3 in the response. Human NP cells were treated with a range of pH, from that of a non-degenerate (pH 7.4 and 7.1) through to mildly degenerate (pH 6.8) and severely degenerate IVD (pH 6.5 and 6.2). Increasing acidity of pH caused a decrease in cell proliferation and viability, a shift towards matrix catabolism and increased expression of proinflammatory cytokines and pain-related factors. Acidic pH resulted in an increase in ASIC-3 expression. Importantly, inhibition of ASIC-3 prevented the acidic pH induced proinflammatory and pain-related phenotype in NP cells. Acidic pH causes a catabolic and degenerate phenotype in NP cells which is inhibited by blocking ASIC-3 activity, suggesting that this may be a useful therapeutic target for treatment of IVD degeneration.

A leading cause of disability is low back pain, affecting around 632 million people globally^1^ and costing the UK economy an estimated £12 billion per annum^2^. The causes of back pain are multifactorial, including genetic predisposition^3^,^4^, lifestyle^5^, as well as mechanical injury^6^, but a significant proportion is associated with degeneration of the intervertebral disc (IVD)^3^,^7^,^8^. The IVD is connected to the superior and inferior vertebral bodies via the cartilaginous endplates (CEP) which, in addition to anchoring the disc within the spine, functions to allow the flow of nutrients and metabolites into and out of the avascular disc, respectively.

The bidirectional flow of nutrients and metabolites is important for the maintenance of the IVD microenvironment, which can be considered as a relatively hostile cellular niche, with large nutrient and metabolite concentration gradients existing across the disc (lower glucose and oxygen in the centre compared to the periphery of the disc) due to cells being as far as 8 mm from a direct blood supply^9^. Low levels of oxygen within the disc results in mainly anaerobic cellular respiration, leading to lactate production as a by-product of glycolysis, and acidification of the central NP region^10^. During ageing and/or degeneration of the disc this bidirectional flow of nutrients and metabolites reduces^11^,^12^,^13^,^14^,^15^,^16^,^17^, leading to an accumulation of lactic acid in the centre of the disc and a lowering of the pH^9^. The pH of IVDs has been shown to range from pH 7.1 in healthy discs^18^, down to values of 6.5 and even 5.7 in severely degenerated discs^19^,^20^. Although the effect of acidic pH on the gene expression of human NP cells has not yet been reported, low pH similar to that found within a degenerate IVD, has been reported to have a significant impact on bovine disc cells, with a reduction in cell viability^21^, proteoglycan and collagen synthesis^22^, but no change in expression of active metalloproteinases (MMPs) in response to low pH^23^, suggesting a shift towards matrix catabolism. Additionally, the expression of NP associated genes (aggrecan, types I and II collagens and matrix degrading enzymes) by mesenchymal progenitor cells derived from the bone marrow of rats^24^,^25^, rat and human adipose tissue^26^,^27^ and rat IVDs^26^ and exposed to differing pH conditions has been demonstrated. Interestingly, all studies reported a decrease in the expression of matrix-associated genes, with increases in gene expression of matrix degrading enzymes following exposure to acidic pH^24^,^25^,^26^,^27^.

During IVD degeneration there is an upregulation of proinflammatory cytokines, including IL -1β^28^,^29^, IL -6^30^, IL -17^30^ and TNFα^31^,^32^, which drive the catabolic cascades associated with the disease. What causes the initial increase in proinflammatory cytokines remains an important aspect of IVD pathology that is not fully understood. Neurotrophic factors, including nerve growth factor (NGF) and brain-derived neurotrophic factor (BDNF), are also increased in degenerate discs^33^,^34^,^35^. Evidence that nerve ingrowth occurs in painful degenerate discs^36^ and studies demonstrating that conditioned medium from degenerate IVD cells promotes increased neurite outgrowth in nerve cells^37^, suggests a role for these factors in nociception associated with IVD degeneration. Acidic pH has been linked to back pain, with Nachemson *et al.* recording reduced intradiscal pH in patients suffering with painful IVD degeneration when compared to asymptomatic patients^20^ and lactic acid found to be a marker for painful degenerate discs^38^. However, whether acidic pH can directly cause the increase in the pro-inflammatory and pain-related factors observed during IVD degeneration still remains to be elucidated.

Acid sensing ion channels (ASICs) are expressed by disc cells and their expression (ASIC -1, -2, -3 and -4) increases with degeneration, suggesting a role for these receptors in pH sensing within the IVD^39^. Furthermore, ASIC proteins have been suggested to promote NP cell survival when cells are cultured in an acidic and hyperosmotic medium, making ASIC proteins strong candidates for NP cell pH sensing^40^.

We hypothesised that low pH, (caused *in vivo* by lactic acid accumulation as a result of glycolysis and diminished diffusion coefficients), causes many of the cellular and molecular changes observed during IVD degeneration, including elevated proinflammatory cytokines and neurotrophins, reduced expression of matrix-associated molecules, increased expression of matrix degrading enzymes and increased cell death. Our aim was to assess the role of pH in the pro-inflammatory, catabolic and neurogenic response of NP cells and to investigate the involvement of ASIC -3, including its potential as a therapeutic target, in this response.

## Results

### Low pH inhibits cell proliferation and reduces viability

**Figure 1:**
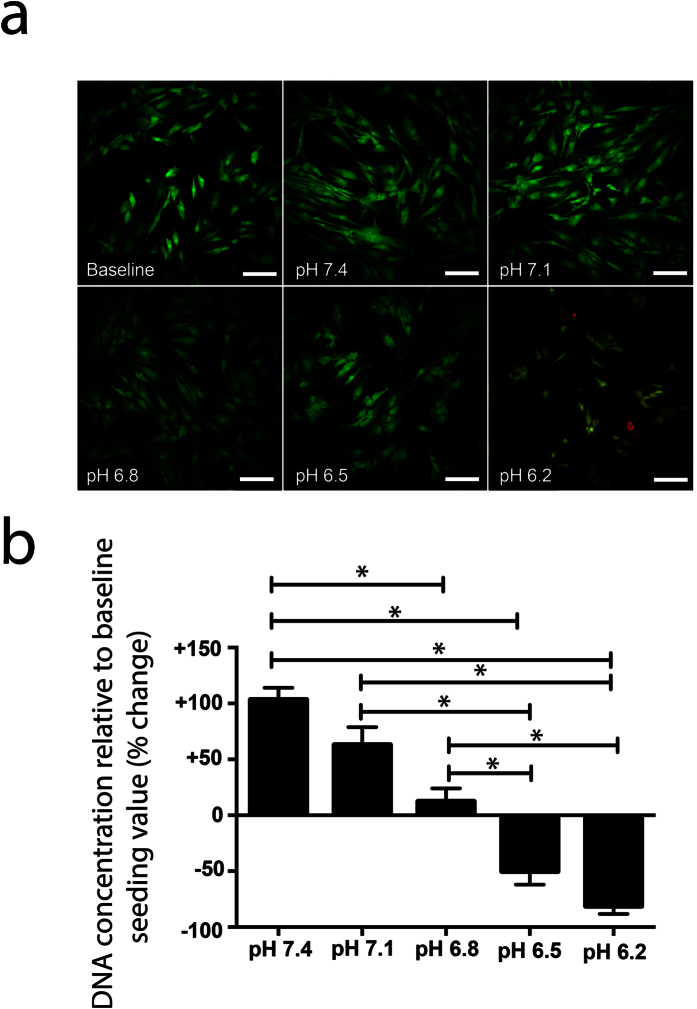
Acidic pH reduces NP cell proliferation and increases cell death. NP cells (n = 3) were cultured in DMEM medium with a pH of 7.4, 7.1, 6.8, 6.5 and 6.2, for 0 days (baseline control) or 7 days and (**a**) Stained with LIVE/DEAD viability stain. Viable and non-viable cells stained green and red, respectively. (**b**) To assess proliferation and/or cell death, a Pico Green assay was used to quantify the total amount of DNA from cells cultured at different pH and normalised to baseline control (0 day of culture at pH 7.4). *Indicates p ≤ 0.05. Scale bar represents 100 μm.

NP cells cultured for 7 days in DMEM at pH 7.4, 7.1 or 6.8 retained high cell viability (indicated by green stained cells without the presence of red nuclei), with no apparent cell death (Fig. 1a). The NP cells cultured at pH 7.4 and 7.1 proliferated when compared to day 0 baseline control, while cells at pH 6.8 appeared quiescent (no difference in amount of DNA harvested at day 7 or baseline) (Fig. 1b). NP cells cultured for 7 days at pH 6.5 or 6.2 had reduced cell viability, as shown by increased numbers of red stained cells (specifying non-viable cells) and reduced amounts of DNA compared to the control baseline samples (Fig. 1a,b). pH 6.5 was used as the lowest pH treatment in all further experiments as the amount of cell death was too great when using medium at pH 6.2.

### Low pH drives expression of inflammatory cytokines and pain-related factors

**Figure 2:**
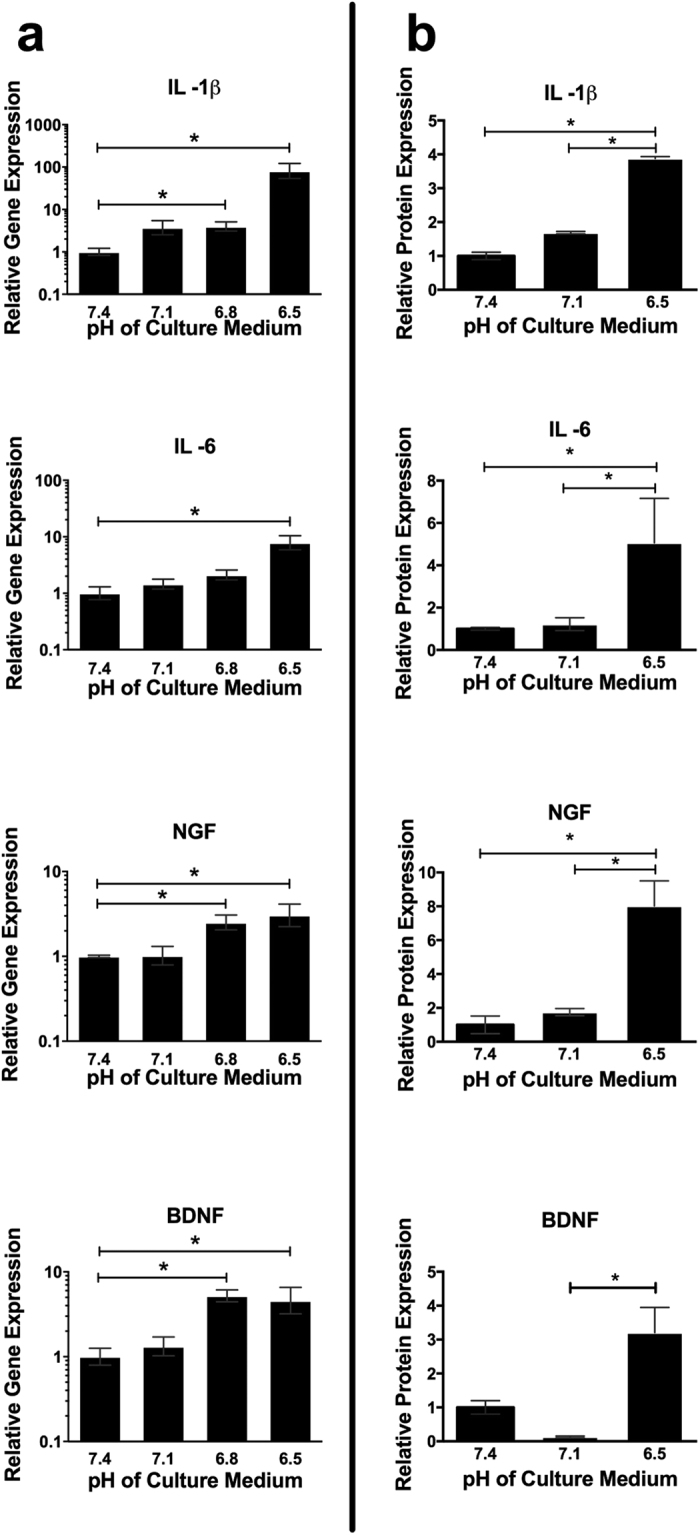
Acidic pH induces increases in proinflammatory and pain-related markers in NP cells. (**a**) Gene expression of IL -1β, IL -6, NGF and BDNF relative to MRPL19 by NP cells (n = 3) cultured at pH 7.1, 6.8 and 6.5 for 7 days and normalised to gene expression at pH 7.4. (**b**) Protein expression (determined by ELISA) of IL -1β, IL -6, NGF and BDNF by NP cells (n = 3) cultured for 7 days at pH 7.1 and 6.5, normalised to expression levels at pH 7.4. *Indicate p ≤ 0.05.

Seven days of culture at pH 7.1 did not alter the gene expression of IL -1β, IL -6, NGF or BDNF compared with control. However, at pH 6.8 there was a significant increase in the gene expression of IL -1β (4.0 -fold), NGF (2.5 -fold) and BDNF (5.2 -fold) compared with control. At pH 6.5 the gene expression of IL -1β (81 -fold), IL -6 (7.8 -fold), NGF (3.0 -fold) and BDNF (4.6 -fold) increased compared to control (Fig. 2a).

The protein expression of IL -1β, IL -6, and NGF was significantly increased (3.9 -fold, 5.1 -fold and 8.0 -fold respectively) at pH 6.5 compared to pH 7.4 and 7.1. BDNF protein was significantly increased (3.2 -fold) at pH 6.5 compared to pH 7.1 (Fig. 2b).

NP cells did not alter their gene expression of TNFα following 7 days of culture in media at any of the pH tested (pH 7.1, 6.8 and 6.5) compared to control pH 7.4 (Supplementary Figure 1).

### Low pH induces a catabolic response in NP cells

**Figure 3:**
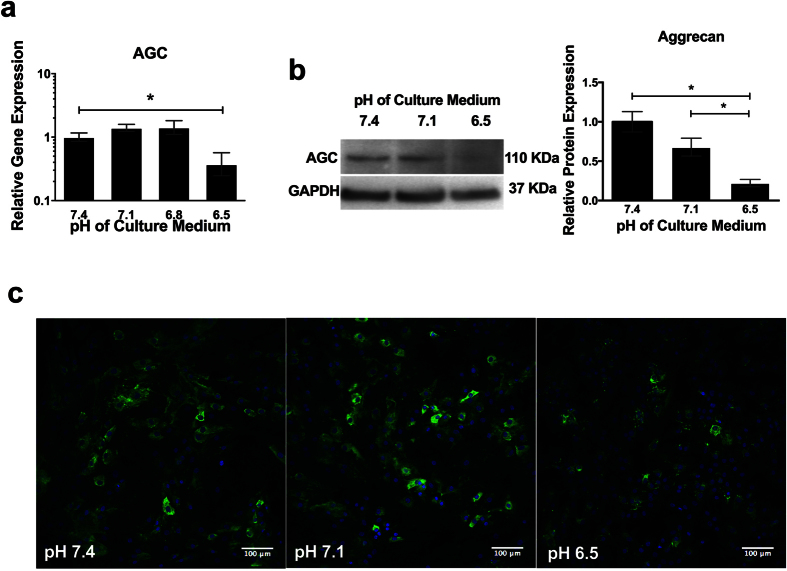
Low pH leads to a decrease in the anabolic protein aggrecan. (**a**) Gene expression of AGC relative to MRPL19 following culture of NP cells (n = 3) at pH 7.4, 7.1, 6.8 and 6.5 for 7 days and normalised to gene expression at pH 7.4. (**b**) Western blot for aggrecan protein expression following culture of NP cells (n = 3) at pH 7.4, 7.1 and 6.5 for 7 days and normalised to GAPDH. *Indicate p ≤ 0.05. (**c**) Immunofluorescence for AGC protein (green) and cell nuclei (DAPI; blue) of NP cells cultured at pH 7.4, 7.1 and 6.5.

**Figure 4:**
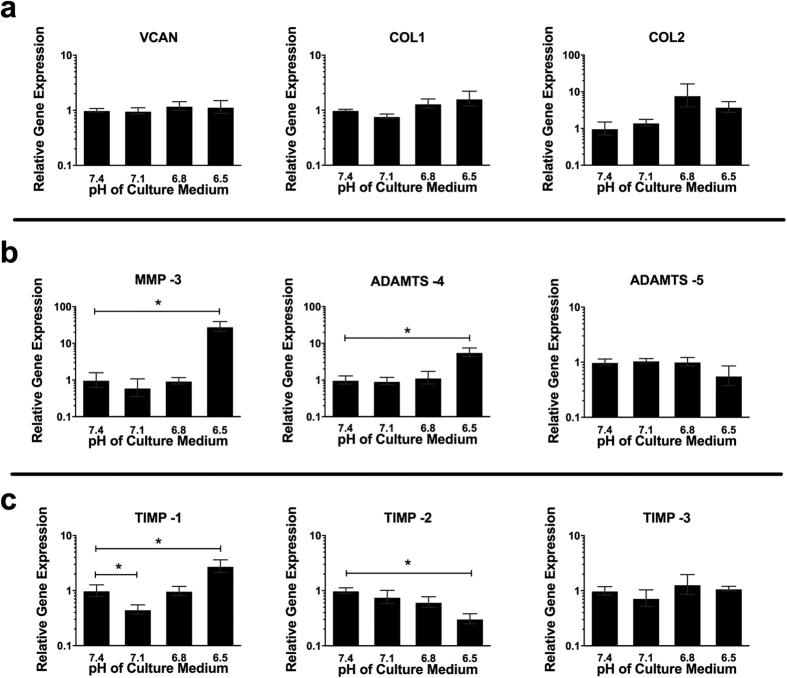
Acidic pH induces a more catabolic phenotype in NP cells. Gene expression of (**a**) matrix proteins, including VCAN, COL1 and COL2A1, (**b**) matrix degrading enzymes, including MMP-3, ADAMTS-4 and -5, and TIMPs-1, -2 and -3, by NP cells (n = 3) cultured at pH 7.1, 6.8 and 6.5 for 7 days and normalised to MRPL19 and gene expression at pH 7.4. *Indicate p ≤ 0.05.

Culture at pH 6.5 for 7 days resulted in a significant decrease in the relative gene expression of AGC (2.7 -fold) (Fig. 3a). This decrease in AGC also occurred at the protein level, with a 4.5 -fold decrease in AGC protein compared to levels at pH 7.4 (Fig. 3b). Immunofluorescence showed AGC to be localised to the cytoplasm of NP cells and the level of immunofluorescent staining was reduced at pH 6.5 compared to pH 7.4 and 7.1 (Fig. 3c). There was no significant change in VCAN, COL1 or COL2 gene expression following treatment at different pH values (Fig. 4a). Acidic pH (pH 6.5) resulted in a significant increase in MMP -3 (28.7 -fold) and ADAMTS -4 (5.7 -fold) gene expression compared to pH 7.4. There was no significant change in ADAMTS -5 gene expression with altered pH. TIMP -1 gene expression decreased significantly at pH 7.1 (2 -fold) compared to pH 7.4, showed no change at pH 6.8 and increased significantly at pH 6.5 (2.8 -fold) (Fig. 4c). The expression of TIMP -2 remained similar to control levels at pH 7.1 and 6.8, but decreased significantly at pH 6.5 (3.3 -fold) (Fig. 4c). TIMP -3 showed no change in expression at any of the pH tested (Fig. 4c).

### Low pH induces changes in expression of acid-sensing ion channel -3 (ASIC-3)

**Figure 5:**
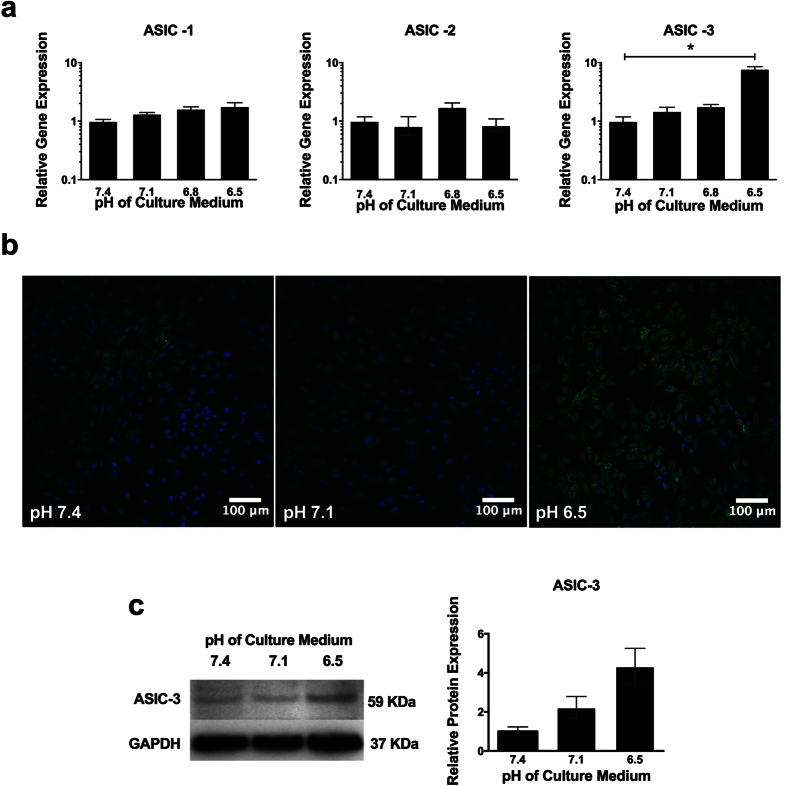
Low pH regulates acid-sensing ion channels (ASICs) in NP cells. (**a**) Gene expression of ASICs-1, -2 and -3 by NP cells (n = 3) cultured at pH 7.1, 6.8 and 6.5 for 7 days and normalised to MRPL19 and gene expression at pH 7.4. (**b**) Immunofluorescence for ASIC -3 (green) and cell nuclei (DAPI; blue) in NP cells cultures at pH 7.4, 7.1 and 6.5 for 7 days. (**c**) Western blot for ASIC-3 protein expression in NP cells (n = 3) cultured at pH 7.1 and 6.5 for 7 days relative to GAPDH and protein expression to pH 7.4. *Indicate p ≤ 0.05.

The gene expression of ASIC -1 and -2 remained unchanged at pH 7.1, 6.8 and 6.5 compared to control (Fig. 5a). ASIC -3 gene expression was unchanged at pH 7.1 and 6.8, but showed a significant increase at pH 6.5 (7.8 –fold (Fig. 5a)). Immunofluorescence showed ASIC -3 staining of NP cells to be cytoplasmic with increased immunofluorescence at pH 6.5 compared to pH 7.1 and 7.4 (Fig. 5b). Western blot analysis of ASIC -3 showed an increase at pH 6.5 compared to pH 7.4 (p = 0.06) (Fig. 5c).

### pH-dependent regulation of IL -1β IL -6, NGF and BDNF protein expression is mediated through the involvement of acid-sensing ion channel (ASIC) -3

**Figure 6:**
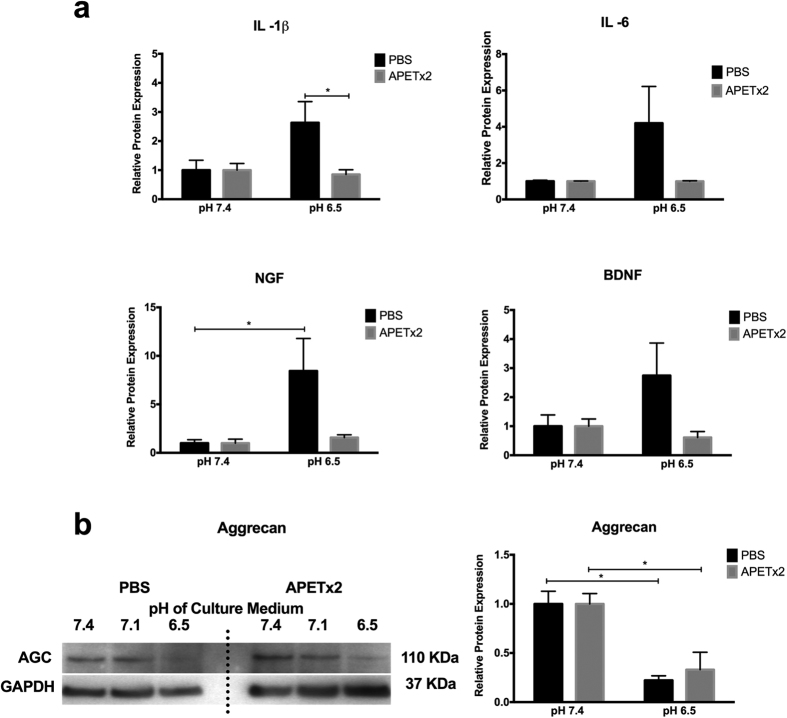
Inhibition of ASIC-3 prevents the pro-inflammatory/pain-related neurogenic response in NP cells exposed to an acidic environment. Expression of proteins assessed using (**a**) ELISA for pro-inflammatory cytokines IL -1β and IL -6, as well as the pain-related neurogenic factors NGF and BDNF, and assessed using (**b**) western blot for aggrecan protein expression by NP cells (n ≥ 3) treated with carrier alone (PBS) or APETx2 (inhibitor of ASIC-3) at 100 nM cultured at pH 7.4 and 6.5 for 7 days and normalised to protein expression levels at pH 7.4. *Indicate p ≤ 0.05.

Treatment of NP cells with 100 nM APETx2 did not influence the amount of proliferation or cell death observed at different pH values (Supplementary Figure 2). NP cells responded to differences in the pH of the culture medium as reported earlier (Fig. 1). NP cells cultured at pH 6.5 increased their protein expression of IL -1 β (2.6 -fold, p = 0.08), IL -6 (4.2 -fold, p = 0.09), NGF (8.4 -fold, p ≤ 0.05) and BDNF (2.7 -fold, p = 0.3), although significance was reached with NGF only (Fig. 6a). Importantly, treatment of NP cells with 100 nM APETx2 (ASIC -3 specific inhibitor) prevented the increase in IL -1β (significance was reached for protein recorded at pH 6.5 between PBS and inhibitor) and IL -6, NGF and BDNF proteins following culture for 7 days at pH 6.5 (Fig. 6a). AGC protein decreased significantly (4.5 -fold) at pH 6.5 compared to pH 7.4. Treatment of NP cells with 100 nM APETx2 did not prevent the acidic pH –induced decrease in AGC protein expression (3 –fold significant decrease at pH 6.5 with the inhibitor) (Fig. 6b).

## Discussion

The pH within the IVD is lower than that in most tissues within the body and this acidic pH decreases further during the onset of ageing and degeneration^19^. Acidic pH has been linked to painful discs^41^ and shown to promote matrix catabolism^21^,^22^,^23^, suggesting low pH may contribute to the cellular and molecular processes observed in IVD degeneration. Whether low pH is the cause of the other phenotypic changes observed during disc degeneration (increased cell death and increased proinflammatory cytokines and neurotrophins) has, until now, remained unknown. This is the first study to show that acidic pH, similar to that reported within degenerate IVDs, results in a proinflammatory, neurotrophic, and catabolic response in human NP cells, features which have been observed during human IVD degeneration.

Human NP cells proliferated in medium at pH 7.4, and 7.1, did not divide at pH 6.8, and showed cell death at pH 6.5 and 6.2. Bovine NP cells have been shown to have reduced viability at pH 6.2; however, this decrease was only slight (about 5% less viable cells than pH 7.4) unless combined with the removal of glucose from the media, at which point cell viability decreased by up to 20%^21^. The longer culture time used in this study (7 days compared with only 1 day) may suggest reasons for the more severe effects on cell viability presented here. Our data does, however, suggest that acidic pH similar to that found in degenerate discs may cause cell death and contribute to the reduced cell numbers observed during IVD degeneration.

Culture of NP cells in acidic pH led to an increase in the proinflammatory cytokines IL -1β and IL -6, but no change in TNFα expression. Proinflammatory cytokine expression, including TNFα, IL -6 and in particular IL -1β expression, has been shown to be upregulated in degenerate IVDs^28^,^29^,^30^, but the initiator for this increase has remained unknown. Our data suggests that the low pH found within degenerate IVDs may be an initiating factor, resulting in increased cytokine production by disc cells. IL -1β has been suggested previously to be the major proinflammatory cytokine involved in the degenerative processes observed during IVD degeneration, with IL -1β treatment of intact disc tissue having a more potent catabolic response (increased expression of active MMPs) than treatment with TNFα^29^. Furthermore, IL -1β, and not TNFα, may act as a master regulator by controlling the expression of other cytokines and downstream catabolic factors, as recently reported by Phillips *et al.*^42^. We found that culture of NP cells at acidic pH did not alter the expression of TNFα, suggesting that TNFα may have less of a role as an initiator in the process of IVD degeneration.

Culture of NP cells at acidic pH also led to increased expression of the neurotrophic factors NGF and BDNF. Degenerate IVDs have previously been shown to have greater expression of NGF^35^,^43^ and BDNF^33^ and expression of both of these neurotrophins is thought to be driven by IL -1β, IL -6 and TNFα^43^,^44^,^45^,^46^. However, to our knowledge, this is the first time that low pH has been shown to upregulate NGF and BDNF expression in IVD cells. Neurite ingrowth has been shown to occur in painful and degenerated IVDs^36^. Increased production of neurotrophins may be responsible for the in-growth of nerve fibres into the degenerate disc and our data suggests that acidic pH may be a contributing factor in this process.

NP cells cultured in acidic pH decreased their expression of aggrecan, suggesting that acidic pH may be involved in the loss of aggrecan observed during IVD degeneration. In addition to a decrease in aggrecan (one of the major extracellular matrix proteins of the disc), the expression of matrix degrading enzymes was increased, suggesting a shift in cell phenotype towards that of matrix catabolism. TIMP expression showed differential responses to acidic pH dependent on the TIMP type, with TIMP -1 increasing, TIMP -2 decreasing and TIMP -3 remaining unaffected by acidic pH. Although the targets of the different TIMPs investigated are relatively broad, some TIMPs have heightened specificity for particular enzymes. TIMP -1, which showed an increase with acidic pH has an affinity for MMP -3, which also increased in response to low pH, suggesting a cellular compensation mechanism to prevent over activity of MMP -3^47^. TIMP -2, which decreased in response to acidic pH, has been shown to bind to MMP -9^48^, suggesting an imbalance could occur during acidic pH conditions leading to increased MMP -9. TIMP -3 has the broadest specificity and has been shown to bind to and inhibit targets as diverse as the ADAMTSs, including ADAMTS -4, again suggesting the possibility for imbalance at low pH between ADAMTS -4 (increased at acidic pH) and TIMP -3 (no change in expression levels) expression^49^. This pattern of decreased anabolic and increased catabolic factors has been demonstrated in cells derived from degenerate tissue, and in cells treated with proinflammatory cytokines, including IL -1β, IL -6 and TNFα^28^,^50^,^51^,^52^. Our study suggests that the low pH associated with IVD degeneration may initiate the shift in matrix homeostasis towards that of increased catabolism, perhaps directly or through the involvement of cytokines (e.g. IL -1β or IL -6).

In order for NP cells to respond to changes in extracellular pH they must express the appropriate molecular machinery in order to sense proton concentrations. NP cells have been shown to express ASICs -1, -2, -3 and -4 and their expression is increased with degeneration^39^, while ASIC receptor inhibition leads to enhanced apoptosis and NP cell death (41). Our study shows for the first time differential regulation of ASICs in NP cells treated with low pH. We identified that ASIC -1 and -2 gene expression remained unchanged, whereas ASIC -3 gene and protein levels increased upon culture of NP cells at acidic pH, suggesting an important role for ASIC -3 in pH-dependent response of NP cells. Interestingly, when a specific inhibitor was used against ASIC -3 (chosen as a target due to its induction following acidic pH), acidic pH-induced increases in IL -1β IL -6, NGF and BDNF protein expression were prevented, suggesting a role for ASIC -3 in the pro-inflammatory and neurogenic response of NP cells to low pH. ASIC -3 has been previously shown to be involved in the pain response of rats following experimentally induced osteoarthritis of their knee joints. Repeated injections of APETx2 reduced the amount of osteoarthritic changes observed and reduced the pain response, with the authors proposing APETx2 as a potential therapeutic agent against osteoarthritis^53^. ASIC -3 may therefore be a suitable therapeutic target for prevention of acidity-driven induction of pro-inflammatory and pain-related factors by NP cells during IVD degeneration, with APETx2 a potential therapeutic agent for treatment of painful IVD degeneration. However, at the concentrations used here APETx2 was unable to prevent the decrease in aggrecan following treatment of NP cells with acidic pH. Similar observations have been reported in a rat osteoarthritis model, where treatment of osteoarthritic knees using APETx2 reduced pain, but was unable to prevent the loss of proteoglycans^53^. Thus the inability of APETx2 to prevent the acidity driven decrease in aggrecan expression suggests that an additional biological factor may be required in parallel with APETx2 treatment, in order to restore IVD composition and function. One approach to restore aggrecan content may be to combine APETx2 treatment with a cell-based therapy, such as GDF6-differentiated adipose stem cells, which we have previously shown results in enhanced aggrecan synthesis and production of an NP-like matrix^54^.

A potential limitation of the use of APETx2, and many other biologically active molecules, is its relatively short half-life (about 2.8 hours when exposed to trypsin, although this half-life may be greater when injected into a degenerate IVD)^55^. It may, however, be possible to modify the half-life of APETx2, which could improve its therapeutic potential. For example, Jensen *et al.* found that the half-life of APETx2 could be increased by about 20–30 times when amino acid residues were added to the C- and N- terminals^55^. The authors found however, that the stabilisation of APETx2 resulted in diminished ASIC-3 inhibition, suggesting that other peptide modifications may be necessary in order to retain peptide activity while increasing stability^55^.

We believe the findings presented here are important to the field of IVD degeneration and could aid in its treatment. However, it is important to acknowledge that as this study was conducted *in vitro*, NP cells may respond differently *in vivo*. Disc cells are not solely exposed to a low pH environment, but are challenged by many other microenvironmental conditions, including changes in osmolarity, hypoxia and glucose concentrations. Considering the effect of acidic pH in isolation on NP cell phenotype is useful in order to remove complicating factors from the experimental setup. Future studies will also need to evaluate the entire plethora of microenvironmental factors present within the degenerate IVD. Furthermore, it will also be important to assess the activity of matrix degrading enzymes, in addition to changes in expression level, and to gain a fuller understanding of the temporal and molecular cascades which result in aberrant cell function. Together such studies would provide a more detailed understanding of the NP cell behaviour in response to the degenerate IVD niche.

To our knowledge this is the first study to show that low pH (similar to that found within degenerate IVDs) promotes the aberrant cellular phenotype observed during IVD degeneration, including increased cell death, increased pro-inflammatory cytokine and neurotrophin expression, reduced matrix protein expression and increased matrix degrading enzyme expression. Proinflammatory cytokines have been known to play a major role in the catabolic cascades observed during IVD degeneration, but the reason for their high levels of expression have remained largely unknown. We provide evidence that acidic pH, likely a result of lactate accumulation due to diminished diffusion coefficients, can directly cause cell death and induction of proinflammatory cytokines and neurogenic factors, leading to matrix catabolism. Our study suggests a role for ASIC -3 in acidity sensing and downstream cellular responses in NP cells. Further work is needed in order to fully understand the mechanisms involved but ASIC -3 may represent a useful target for the treatment of acidity-induced disc degeneration.

## Materials and Methods

### IVD Tissue

Human IVD tissue was collected from patients undergoing lumbar spinal surgery for degenerative disc disease (DDD) (one male and two female with a mean age of 50.3 years, SD of 4.16 years) or from cadavers (within 18 hours of death) with patient or relatives written informed consent and Research Ethics Committees approval (National Research Ethics Service Committee North West), with all methodology performed in accordance with the Committee’s guidelines (three males of mean age 45.3 years, SD of 25.3 years). Tissue was processed for cell extraction and representative samples of all tissues containing intact AF and NP regions were formalin-fixed, paraffin-embedded and sections histologically graded as previously reported^56^. All surgical samples were histologically graded as level 8 (classed as degenerate tissue), while cadaveric tissue was histologically graded as 0–3 (non-degenerate) and were obtained from individuals with no documented history of back pain.

### Isolation and culture of NP cells

NP tissue (excluding outer and inner AF) was separated from the IVD within 24 hours of death or surgical removal and finely minced prior to enzymatic digestion as previously reported^56^. NP cells were cultured in standard medium [Dulbecco’s modified Eagle’s medium (DMEM) with glucose 4.5 g/L, L-alanyl-glutamine (Sigma) containing 100 mM sodium pyruvate, 10 μM ascorbate-2-phosphate, 250 ng/mL amphotericin, 100 U/mL penicillin, 100 μg/mL streptomycin (Invitrogen) and 10% foetal calf serum (Life Technologies)] and expanded in monolayer with medium changed every 2–3 days. Sub-confluent NP cells with passage numbers of ≤4 were trypsinised (Life Technologies) and seeded into 6-well tissue culture plates at a density of 5 × 10^4^ cells/mL in 2 mL of standard medium and allowed to adhere for 24 hours. Media was then changed to pH-modified DMEM medium (pH 7.4, 7.1, 6.8, 6.5 and 6.2) and cells cultured for 7 days with and without an inhibitor against ASIC-3 (100 nM APETx2 (R&D Systems)^57^). APETx2, a peptide first purified from the sea anemone *Anthopleura elegantissima,* is a potent and selective inhibitor of ASIC-3. The concentration of APETx2 was chosen based on published IC_50_ values, which were found to lead to specific ASIC-3 inhibition (concentrations of APETx2 above 100 nM were found to also inhibit heteromeric ASIC-2b + 3)^57^, and the ability of APETx2 (tested at 1, 10, and 100 nM concentrations) to prevent the acidic pH –induced increase in IL -6 gene expression (Supplementary Figure 3).

### Preparation of pH modified medium

Basal media was prepared from DMEM/F-12 powdered medium without sodium bicarbonate (Fisher Scientific) and the following supplements added: 50 μg/mL ascorbic acid, 250 ng/mL amphotericin, 100 U/mL penicillin, 100 μg/mL streptomycin (Invitrogen) and 10% foetal calf serum (Life Technologies). The amount of sodium bicarbonate required was calculated using the Henderson-Hasselbalch equation, which for the purpose of sodium bicarbonate buffered culture medium, can be expressed as pH = 6.1 + log (52(mg/ml NaHC03/%CO2)-1)^58^. HCl and NaOH were used to pH the medium to the desired values (pH 7.4, 7.1, 6.8, 6.5 and 6.2) and the medium allowed to equilibrate overnight in a 5% CO_2_ incubator at 37 °C. The pH of the medium was confirmed a second time and the medium sterilised using a 0.04 μm filter and stored at 4 °C until needed (medium not stored for longer than 1 week).

### Viability assay

NP cells in 6-well culture plates were stained with LIVE/DEAD® viability stain (Life Technologies L3224) as per the manufacturer’s instructions and imaged using a Leica SP5 upright confocal microscope with dipping lens. Green staining identified viable cells, while non-viable cells were red, or had red nuclei with green cytoplasm.

### Proliferation assay

NP cells cultured for 0 days (baseline control at pH 7.4) and 7 days in pH modified medium were washed in PBS and lysed using 3 x freeze/thaw cycles in PBS. The amount of DNA from each well was quantified using a Pico Green assay kit, as per manufacturer’s instructions. A standard curve was generated using lambda control DNA and fluorescence detected using a fluorescent microplate reader and compared between samples cultured at different pH.

### Quantitative real time PCR (QRT-PCR)

Total RNA was extracted using TriReagent (Ambion) according to the manufacturer’s instructions and samples treated with DNase I (Ambion) as previously reported^56^. RNA quality and quantity were determined using the Nanodrop ND-1000 Spectrophotometer (Nanodrop Technologies) and 1** **μg of RNA reverse transcribed using the High Capacity Reverse Transcription Kit (Applied Biosystems). QRT-PCR was performed in triplicate using LuminoCt qPCR ReadyMix (Sigma) with primers and probes (Sigma, unless otherwise stated) for mitochondrial ribosomal protein L19 (MRPL19) forward primer (F)-CCACATTCCAGAGTTCTA, reverse primer (R)-CCGAGGATTATAAAGTTCAAA, probe (P)-CAAATCTCGACACCTTGTCCTTCG; acid sensing ion channels ((ASICs)-1 F-CTGTCCATGGTCAAGATC, R-GGATGTTCTCCCCTATGTA, P-AGCCTCAGCCAAGTACCTGG; ASIC-2 F-GGTGATATTGGTGGTCAG, R-GTCACAAGTACTCACATTC, P-TTGTTCATTGGTGCTAGTATCCTTACA; ASIC-3 F-GAGGGACAATGAGGAGAC, R-CCAGCTGATCGATGATGG, P-CTCCTCCTGGCTGTGGATCTG; tumour necrosis factor (TNF) –α F-AGGCAGTCAGATCATCTTC, R-CTGGTTATCTCTCAGCTCC, P-CCGAGTGACAAGCCTGTAGC; interleukin (IL) -1β (Taq Man assay HS01555410_m1 (Applied Biosystems)) and -6 F-CTGGATTCAATGAGGAGAC R-ACTGGATCAGGACTTTTG P-CTGGCTTGTTCCTCACT; nerve growth factor (NGF) (Taq Man assay HS00171458_m1 (Applied Biosystems)), brain-derived neurotrophic factor (BDNF) (Taq Man HS00542425_s1 (Applied Biosystems)), aggrecan (AGC) F-GGCTTCCACCAGTGTGAC, R-GTGTCTCGGATGCCATACG, P-TGACCAGACTGTCAGATACCCCATCCA; versican (VCAN) F-TCCCTCACTGTGGTCAAG, R-GTGTGTACCTGCTGGTTG, P-AAACACAACCCCATCCACAGTCAGT; type I and II collagens (COL1A1) F-TCAGCTTTGTGGATACGC, R-CTGGGCCTTTCTTACAG, P-CAGTAACCTTATGCCTAGCAACATGC; and (COL2A1) (Taq Man assay HS00264051_m1 (Applied Biosystems)); matrix metalloproteinase (MMP)-3 F-GTGGAGTTCCTGATGTTG, R-GCATCTTTTGGCAAATCTG, P-AATTCACAATCCTGTATGTAAGGT; a disintegrin and metalloproteinase with a thrombospondin type 1 motif (ADAMTS)-4 F-TCAGGAAATTCAGGTACG, R-CGTGTATTCACCATTGAG, P-CATAGGAGCCATCTGGC; ADAMTS-5 F-CGCTTAATGTCTTCCATCCTTA, R-GGATCTGCTTTCGTGGTAG, P-CAGCAAACAGTTACCATGGCCATCATC; and tissue inhibitor of metalloproteinases (TIMPs)-1 F-GACACCAGAGAACCCA, R-GACGAGGTCGGAATTG, P-CTGGCTTCTGGCATCCT; TIMP-2 F-TGCAGATGTAGTGATCAG, R-TGCCATAAATGTCGTTTC, P-ACTTCCTTCTCACTGAC; TIMP-3 F-GGTTGTAACTGCAAGATCAA, R-CAGAGACACTCGTTCTTG, P-CCTGCTACTACCTGCC. Data was analysed using the 2^−∆∆Ct^ method^56^,^59^ and normalised to the endogenous control gene MRPL19 and pH 7.4 control.

### Enzyme-linked immunosorbent assay (ELISA)

The expression of soluble factors from NP cells cultured at different pH was assessed using ELISA as described by the manufacturer. Briefly, samples were cleared of cell debris and added in triplicate to ELISA plates, each with specific antibodies against NGF (Abcam ab99986), BDNF (Abcam ab99978), IL -1β (Abcam ab100562) and IL -6 (Qiagen). TNFα protein expression was not assessed as no significant change was observed at the gene level with differing pH values. Plates were kept at 4 °C overnight and then washed 3 x in PBS before biotinylated detector antibodies were added. After incubation at room temp for 1 hour, 96-well plates were washed again 3 x PBS and HRP-streptavidin solution added for 45 mins. TMB substrate reagent was then added and incubated for 30 mins in the dark followed by the addition of stopping solution. The absorbance was assessed using a microplate reader with filter for 450 nm. The amount of protein present within each sample (pH 7.1 and pH 6.5) was compared back to the control (pH 7.4) and fold change in protein reported.

### Western blot analysis

Total protein was extracted using radioimmunoprecipitation assay (RIPA) lysis buffer (50 mM Tris-HCL, 1% Triton-X (Sigma), 0.25% Sodium deoxycholate (Sigma), 150 mM NaCl (Sigma), Halt™ Protease/Phosphatase inhibitor Single-Use cocktail (Thermo Scientific)) at 4 °C with frequent agitation for 20 minutes, as previously described^60^. Cell lysates were cleared of insoluble debris by centrifugation at 12,000× g for 10 minutes at 4 °C and protein quantified using the Pierce^®^ BCA Protein Assay according to the manufacturer’s instructions. Whole cell lysates were reduced by incubation with 5X Laemmli reducing buffer (250 mM Tris-HCl (pH 6.8), 25% Glycerol (Sigma), 10% sodium dodecyl sulphate (SDS), 500 mM Dithiothreitol (DTT) (Sigma), 0.05% Bromophenol blue dye (Sigma)) at 95 °C for 10 minutes. Equal amounts of cell lysates (5 μg/well) were separated using 10% SDS – polyacrylamide gel electrophoresis (SDS – PAGE) and protein transferred to polyvinylidene fluoride (PVDF) membranes (GE Healthcare). PVDF membranes were blocked with 5% (w/v) non-fat milk (Alcafe) in Tris-buffered saline (50 mM Tris, 139 mM NaCl, pH 7.6) containing 0.1% Tween 20 (Sigma) (TBS-T) overnight at 4 °C with constant agitation. Membranes were then washed with TBS-T and incubated with 5% (w/v) non-fat milk in TBS-T with anti- aggrecan (1:250) (AbD Serotec MCA1454G) or anti- ASIC -3 antibody (1:300) (Abcam ab49333) for 2 hours at room temperature with constant agitation. Membranes were washed 3 times with TBS-T and incubated with 5% (w/v) non-fat milk in TBS-T with horseradish peroxidise-conjugated secondary antibody (goat anti- rabbit (R&D Systems; HAF008) or donkey anti-mouse (R&D Systems; HAF018), respectively, for 1 hour at room temperature. Membranes were washed 3 times with TBS-T, developed using ECL chemiluminescent reagent (PerkinElmer) according to the manufacturer’s protocol and exposed to photographic film (Blue XB, Kodak). Following 3 washes with TBS-T, the membranes were incubated with 5% (w/v) non-fat milk in TBS-T with anti- glyceraldehyde 3-phosphate dehydrogenase (GAPDH) antibody (1:10,000) (Calbiochem CB1001) for 1 hour at room temperature with constant agitation. Membranes were washed 3 times with TBS-T and incubated with 2% (w/v) non-fat milk in TBS-T with horseradish peroxidise-conjugated secondary antibody donkey anti- mouse (R&D Systems; HAF018) for 1 hour at room temperature and protein visualised as described above. The density of each protein band was quantified using a Syngene imaging system, and the ratio of the density of aggrecan or ASIC -3 protein bands to the density of GAPDH protein bands calculated. The relative protein expression calculated at pH 7.1 and 6.5 was then normalised to that at pH 7.4.

### Immunofluorescence

Following 7 days culture in pH-modified DMEM medium cells adhered to 6-well culture plates were washed in PBS and fixed with 4% PFA at room temperature for 10 mins. PFA was removed and cells washed once in PBS before being stored at 4 °C in PBS. Cells were permeabalised in 0.25% Triton-X in PBS (PBS-T) for 5 mins, washed once in PBS and blocked using 1% BSA in PBS-T (blocking buffer) for 30 mins. Blocking buffer was removed, primary antibody for aggrecan (AbD Serotec MCA1454G) or ASIC-3 (Abcam ab49333) added at 1:1000 dilution in blocking buffer and stored at 4 °C overnight. Sample wells were washed 3 x PBS and incubated with secondary antibody 1:2000 in blocking buffer, donkey anti-mouse, or anti-rabbit 488 AlexaFluor (ThermoScientific) for 1 hour at room temperature in the dark. Samples were washed 3 x in PBS and then incubated in blocking buffer with 1:1000 DAPI (ThermoScientific) for 20 mins at room temp in the dark. Samples were washed 3 x in PBS and stored in the dark at 4 °C until imaged using a Leica SP5 upright confocal microscope.

### Statistics

All data were compared using the nonparametric Mann-Whitney U test with p ≤ 0.05 reported as significant.

## Additional Information

**How to cite this article**: Gilbert, H. T. J. *et al.* Acidic pH promotes intervertebral disc degeneration: Acid-sensing ion channel -3 as a potential therapeutic target. *Sci. Rep.* **6**, 37360; doi: 10.1038/srep37360 (2016).

**Publisher’s note:** Springer Nature remains neutral with regard to jurisdictional claims in published maps and institutional affiliations.

## Supplementary Material

Supplementary Information

## References

[b1] Vos T. *et al.* Years lived with disability (YLDs) for 1160 sequelae of 289 diseases and injuries 1990–2010: a systematic analysis for the Global Burden of Disease Study 2010. Lancet 380, 2163–2196, doi: 10.1016/S0140-6736(12)61729-2 (2012). 23245607 PMC6350784

[b2] Maniadakis N. & Gray A. The economic burden of back pain in the UK. Pain 84, 95–103 (2000). 10601677 10.1016/S0304-3959(99)00187-6

[b3] Livshits G. *et al.* Lumbar disc degeneration and genetic factors are the main risk factors for low back pain in women: the UK Twin Spine Study. Ann Rheum Dis 70, 1740–1745, doi: 10.1136/ard.2010.137836 (2011). 21646416 PMC3171106

[b4] Patel A. A., Spiker W. R., Daubs M., Brodke D. & Cannon-Albright L. A. Evidence for an inherited predisposition to lumbar disc disease. J Bone Joint Surg Am 93, 225–229, doi: 10.2106/JBJS.J.00276 (2011). 21266637 PMC3028451

[b5] Lefevre-Colau M. M. *et al.* Frequency and interrelations of risk factors for chronic low back pain in a primary care setting. PLoS One 4, e4874, doi: 10.1371/journal.pone.0004874 (2009). 19287499 PMC2654108

[b6] Magnusson M. L. *et al.* European Spine Society–the AcroMed Prize for Spinal Research 1995. Unexpected load and asymmetric posture as etiologic factors in low back pain. Eur Spine J 5, 23–35 (1996). 8689414 10.1007/BF00307824

[b7] Luoma K. *et al.* Low back pain in relation to lumbar disc degeneration. Spine (Phila Pa 1976) 25, 487–492 (2000). 10707396 10.1097/00007632-200002150-00016

[b8] Peterson C. K., Bolton J. E. & Wood A. R. A cross-sectional study correlating lumbar spine degeneration with disability and pain. Spine (Phila Pa 1976) 25, 218–223 (2000). 10685487 10.1097/00007632-200001150-00013

[b9] Urban J. P. The role of the physicochemical environment in determining disc cell behaviour. Biochem Soc Trans 30, 858–864, doi: 10.1042/ (2002). 12440933 10.1042/bst0300858

[b10] Bibby S. R., Jones D. A., Ripley R. M. & Urban J. P. Metabolism of the intervertebral disc: effects of low levels of oxygen, glucose, and pH on rates of energy metabolism of bovine nucleus pulposus cells. Spine (Phila Pa 1976) 30, 487–496 (2005). 15738779 10.1097/01.brs.0000154619.38122.47

[b11] Roberts S., Menage J. & Eisenstein S. M. The cartilage end-plate and intervertebral disc in scoliosis: calcification and other sequelae. J Orthop Res 11, 747–757, doi: 10.1002/jor.1100110517 (1993). 8410475

[b12] McFadden K. D. & Taylor J. R. End-plate lesions of the lumbar spine. Spine (Phila Pa 1976) 14, 867–869 (1989). 2781398 10.1097/00007632-198908000-00017

[b13] Aoki J. *et al.* End plate of the discovertebral joint: degenerative change in the elderly adult. Radiology 164, 411–414, doi: 10.1148/radiology.164.2.3602378 (1987). 3602378

[b14] Roberts S., Urban J. P., Evans H. & Eisenstein S. M. Transport properties of the human cartilage endplate in relation to its composition and calcification. Spine (Phila Pa 1976) 21, 415–420 (1996). 8658243 10.1097/00007632-199602150-00003

[b15] Urban M. R. *et al.* Electrochemical measurement of transport into scoliotic intervertebral discs *in vivo* using nitrous oxide as a tracer. Spine (Phila Pa 1976) 26, 984–990 (2001). 11317125 10.1097/00007632-200104150-00028

[b16] Battie M. C. *et al.* 1991 Volvo Award in clinical sciences. Smoking and lumbar intervertebral disc degeneration: an MRI study of identical twins. Spine (Phila Pa 1976) 16, 1015–1021 (1991). 1948392

[b17] Kauppila L. I., Penttila A., Karhunen P. J., Lalu K. & Hannikainen P. Lumbar disc degeneration and atherosclerosis of the abdominal aorta. Spine (Phila Pa 1976) 19, 923–929 (1994). 8009350 10.1097/00007632-199404150-00010

[b18] Ichimura K., Tsuji H., Matsui H. & Makiyama N. Cell culture of the intervertebral disc of rats: factors influencing culture, proteoglycan, collagen, and deoxyribonucleic acid synthesis. J Spinal Disord 4, 428–436 (1991). 1810565 10.1097/00002517-199112000-00004

[b19] Diamant B., Karlsson J. & Nachemson A. Correlation between lactate levels and pH in discs of patients with lumbar rhizopathies. Experientia 24, 1195–1196 (1968). 5703005 10.1007/BF02146615

[b20] Nachemson A. Intradiscal measurements of pH in patients with lumbar rhizopathies. Acta Orthop Scand 40, 23–42 (1969). 4312806 10.3109/17453676908989482

[b21] Bibby S. R. & Urban J. P. Effect of nutrient deprivation on the viability of intervertebral disc cells. Eur Spine J 13, 695–701, doi: 10.1007/s00586-003-0616-x (2004). 15048560 PMC3454063

[b22] Ohshima H. & Urban J. P. The effect of lactate and pH on proteoglycan and protein synthesis rates in the intervertebral disc. Spine (Phila Pa 1976) 17, 1079–1082 (1992). 1411761 10.1097/00007632-199209000-00012

[b23] Razaq S., Wilkins R. J. & Urban J. P. The effect of extracellular pH on matrix turnover by cells of the bovine nucleus pulposus. Eur Spine J 12, 341–349, doi: 10.1007/s00586-003-0582-3 (2003). 12883962 PMC3467790

[b24] Wuertz K., Godburn K. & Iatridis J. C. MSC response to pH levels found in degenerating intervertebral discs. Biochem Biophys Res Commun 379, 824–829, doi: 10.1016/j.bbrc.2008.12.145 (2009). 19133233 PMC2652844

[b25] Wuertz K., Godburn K., Neidlinger-Wilke C., Urban J. & Iatridis J. C. Behavior of mesenchymal stem cells in the chemical microenvironment of the intervertebral disc. Spine (Phila Pa 1976) 33, 1843–1849, doi: 10.1097/BRS.0b013e31817b8f53 (2008). 18670337 PMC2567058

[b26] Han B. *et al.* Nucleus pulposus mesenchymal stem cells in acidic conditions mimicking degenerative intervertebral discs give better performance than adipose tissue-derived mesenchymal stem cells. Cells Tissues Organs 199, 342–352, doi: 10.1159/000369452 (2014). 25661884

[b27] Li H. *et al.* Acidic pH conditions mimicking degenerative intervertebral discs impair the survival and biological behavior of human adipose-derived mesenchymal stem cells. Exp Biol Med (Maywood) 237, 845–852, doi: 10.1258/ebm.2012.012009 (2012). 22829705

[b28] Le Maitre C. L., Freemont A. J. & Hoyland J. A. The role of interleukin-1 in the pathogenesis of human intervertebral disc degeneration. Arthritis Res Ther 7, R732–R745, doi: 10.1186/ar1732 (2005). 15987475 PMC1175026

[b29] Hoyland J. A., Le Maitre C. & Freemont A. J. Investigation of the role of IL-1 and TNF in matrix degradation in the intervertebral disc. Rheumatology (Oxford) 47, 809–814, doi: 10.1093/rheumatology/ken056 (2008). 18397957

[b30] Shamji M. F. *et al.* Proinflammatory cytokine expression profile in degenerated and herniated human intervertebral disc tissues. Arthritis Rheum 62, 1974–1982, doi: 10.1002/art.27444 (2010). 20222111 PMC2917579

[b31] Hiyama A., Yokoyama K., Nukaga T., Sakai D. & Mochida J. A complex interaction between Wnt signaling and TNF-alpha in nucleus pulposus cells. Arthritis Res Ther 15, R189, doi: 10.1186/ar4379 (2013). 24286133 PMC3978705

[b32] Weiler C., Nerlich A. G., Bachmeier B. E. & Boos N. Expression and distribution of tumor necrosis factor alpha in human lumbar intervertebral discs: a study in surgical specimen and autopsy controls. Spine (Phila Pa 1976) 30, 44–53; discussion 54 (2005). 15626980 10.1097/01.brs.0000149186.63457.20

[b33] Purmessur D., Freemont A. J. & Hoyland J. A. Expression and regulation of neurotrophins in the nondegenerate and degenerate human intervertebral disc. Arthritis Res Ther 10, R99, doi: 10.1186/ar2487 (2008). 18727839 PMC2575613

[b34] Gruber H. E. *et al.* Brain-derived neurotrophic factor and its receptor in the human and the sand rat intervertebral disc. Arthritis Res Ther 10, R82, doi: 10.1186/ar2456 (2008). 18637190 PMC2575628

[b35] Freemont A. J. *et al.* Nerve growth factor expression and innervation of the painful intervertebral disc. J Pathol 197, 286–292, doi: 10.1002/path.1108 (2002). 12115873

[b36] Freemont A. J. *et al.* Nerve ingrowth into diseased intervertebral disc in chronic back pain. Lancet 350, 178–181 (1997). 9250186 10.1016/s0140-6736(97)02135-1

[b37] Richardson S. M. *et al.* Degenerate human nucleus pulposus cells promote neurite outgrowth in neural cells. PLoS One 7, e47735, doi: 10.1371/journal.pone.0047735 (2012). 23091643 PMC3472988

[b38] Keshari K. R. *et al.* Lactic acid and proteoglycans as metabolic markers for discogenic back pain. Spine (Phila Pa 1976) 33, 312–317, doi: 10.1097/BRS.0b013e31816201c3 (2008). 18303465

[b39] Cuesta A. *et al.* Acid-sensing ion channels in healthy and degenerated human intervertebral disc. Connect Tissue Res 55, 197–204, doi: 10.3109/03008207.2014.884083 (2014). 24432912

[b40] Uchiyama Y. *et al.* Expression of acid-sensing ion channel 3 (ASIC3) in nucleus pulposus cells of the intervertebral disc is regulated by p75NTR and ERK signaling. J Bone Miner Res 22, 1996–2006, doi: 10.1359/jbmr.070805 (2007). 17696763

[b41] Liang C. Z. *et al.* The relationship between low pH in intervertebral discs and low back pain: a systematic review. Arch Med Sci 8, 952–956, doi: 10.5114/aoms.2012.32401 (2012). 23319966 PMC3542485

[b42] Phillips K. L. *et al.* Potential roles of cytokines and chemokines in human intervertebral disc degeneration: interleukin-1 is a master regulator of catabolic processes. Osteoarthritis Cartilage 23, 1165–1177, doi: 10.1016/j.joca.2015.02.017 (2015). 25748081

[b43] Binch A. L. *et al.* Expression and regulation of neurotrophic and angiogenic factors during human intervertebral disc degeneration. Arthritis Res Ther 16, 416, doi: 10.1186/s13075-014-0416-1 (2014). 25209447 PMC4177417

[b44] Abe Y. *et al.* Proinflammatory cytokines stimulate the expression of nerve growth factor by human intervertebral disc cells. Spine (Phila Pa 1976) 32, 635–642, doi: 10.1097/01.brs.0000257556.90850.53 (2007). 17413467

[b45] Lee J. M. *et al.* Interleukin-1beta induces angiogenesis and innervation in human intervertebral disc degeneration. J Orthop Res 29, 265–269, doi: 10.1002/jor.21210 (2011). 20690185

[b46] Murphy P. G. *et al.* Reciprocal actions of interleukin-6 and brain-derived neurotrophic factor on rat and mouse primary sensory neurons. Eur J Neurosci 12, 1891–1899 (2000). 10886330 10.1046/j.1460-9568.2000.00074.x

[b47] Woessner J. F. Jr. Matrix metalloproteinases and their inhibitors in connective tissue remodeling. FASEB J 5, 2145–2154 (1991). 1850705

[b48] Welgus H. G., Jeffrey J. J., Eisen A. Z., Roswit W. T. & Stricklin G. P. Human skin fibroblast collagenase: interaction with substrate and inhibitor. Coll Relat Res 5, 167–179 (1985). 2988853 10.1016/s0174-173x(85)80038-8

[b49] Wayne G. J. *et al.* TIMP-3 inhibition of ADAMTS-4 (Aggrecanase-1) is modulated by interactions between aggrecan and the C-terminal domain of ADAMTS-4. J Biol Chem 282, 20991–20998, doi: 10.1074/jbc.M610721200 (2007). 17470431

[b50] Koshy P. J. *et al.* The modulation of matrix metalloproteinase and ADAM gene expression in human chondrocytes by interleukin-1 and oncostatin M: a time-course study using real-time quantitative reverse transcription-polymerase chain reaction. Arthritis Rheum 46, 961–967 (2002). 11953973 10.1002/art.10212

[b51] Studer R. K., Vo N., Sowa G., Ondeck C. & Kang J. Human nucleus pulposus cells react to IL-6: independent actions and amplification of response to IL-1 and TNF-alpha. Spine (Phila Pa 1976) 36, 593–599, doi: 10.1097/BRS.0b013e3181da38d5 (2011). 21178846

[b52] Wuertz K. & Haglund L. Inflammatory mediators in intervertebral disk degeneration and discogenic pain. Global Spine J 3, 175–184, doi: 10.1055/s-0033-1347299 (2013). 24436868 PMC3854585

[b53] Izumi M., Ikeuchi M., Ji Q. & Tani T. Local ASIC3 modulates pain and disease progression in a rat model of osteoarthritis. J Biomed Sci 19, 77, doi: 10.1186/1423-0127-19-77 (2012). 22909215 PMC3520115

[b54] Clarke L. E. *et al.* Growth differentiation factor 6 and transforming growth factor-beta differentially mediate mesenchymal stem cell differentiation, composition, and micromechanical properties of nucleus pulposus constructs. Arthritis Res Ther 16, R67, doi: 10.1186/ar4505 (2014). 24618041 PMC4060243

[b55] Jensen J. E. *et al.* Cyclisation increases the stability of the sea anemone peptide APETx2 but decreases its activity at acid-sensing ion channel 3. Mar Drugs 10, 1511–1527, doi: 10.3390/md10071511 (2012). 22851922 PMC3407927

[b56] Gilbert H. T., Hoyland J. A. & Millward-Sadler S. J. The response of human anulus fibrosus cells to cyclic tensile strain is frequency-dependent and altered with disc degeneration. Arthritis Rheum 62, 3385–3394, doi: 10.1002/art.27643 (2010). 20617521

[b57] Diochot S. *et al.* A new sea anemone peptide, APETx2, inhibits ASIC3, a major acid-sensitive channel in sensory neurons. EMBO J 23, 1516–1525, doi: 10.1038/sj.emboj.7600177 (2004). 15044953 PMC391081

[b58] Esser P. *pH and pressure in closed tissue culture vessels*, https://www.thermoscientific.com/content/dam/tfs/LPG/LCD/LCDDocuments/Application%26TechnicalNotes/CellCultureVesselsandMicroplates/CellCultureSystems/pHandpressureissuesinclosedcellculturevessels.pdf (2010).

[b59] Livak K. J. & Schmittgen T. D. Analysis of relative gene expression data using real-time quantitative PCR and the 2(-Delta Delta C(T)) Method. Methods 25, 402–408, doi: 10.1006/meth.2001.1262 (2001). 11846609

[b60] Gilbert H. T., Nagra N. S., Freemont A. J., Millward-Sadler S. J. & Hoyland J. A. Integrin - dependent mechanotransduction in mechanically stimulated human annulus fibrosus cells: evidence for an alternative mechanotransduction pathway operating with degeneration. PLoS One 8, e72994, doi: 10.1371/journal.pone.0072994 (2013). 24039840 PMC3764176

