# Supplementary material for: Acidic pH promotes intervertebral disc degeneration: Acid-sensing ion channel -3 as a potential therapeutic target
Source: Sci Rep. 2016 Nov 17;6:37360. doi: 10.1038/srep37360 (PMC5112591; doi:10.1038/srep37360)
Supplement: Supplementary Information [file srep37360-s1.doc]

**Title**: Acidic pH promotes intervertebral disc degeneration: Acid-sensing ion channel -3 as a potential therapeutic target.

**Authors:** Hamish T J Gilbert (Hamish.Gilbert@manchester.ac.uk)

Nathan Hodson (NWH151@student.bham.ac.uk)

Pauline Baird (Pauline.Baird@manchester.ac.uk)

Stephen M Richardson (S.Richardson@manchester.ac.uk)

Judith A Hoyland (Judith.a.hoyland@manchester.ac.uk)

**Supplementary information.**

**Supplementary figure 1:** Acidic pH does not alter the gene expression of TNFα in NP cells. Gene expression of TNFα relative to MRPL19 by NP cells (n=3) cultured at pH 7.1, 6.8 and 6.5 for 7 days and normalised to gene expression at pH 7.4.

**Supplementary figure 2:** Treatment of NP cells with APETx2 does not effect the pH –induced changes in proliferation or cell death following 7 days of culture at different pH. NP cells (n=3) were cultured in DMEM medium with a pH of 7.4, 7.1 and 6.5, for 7 days and a: stained with LIVE/DEAD viability stain. Viable and non-viable cells stained green and red, respectively. Scale bar represents 100 μm. b: To assess proliferation and/or cell death, a Pico Green assay was used to quantify the total amount of DNA from cells cultured at different pH, normalised to 7 days of culture at pH 7.4. * indicates p ≤ 0.05.

**Supplementary figure 3:** Treatment of NP cells with 100nM of APETx2 inhibits the acidity-driven increase in IL-6 gene expression. NP cells (n=3) were treated with a range of APETx2 concentrations (1, 10 and 100 nM) and cultured for 7 days at pH 7.4 or 6.5. The gene expression of IL-6 relative to MRPL19 and normalised to pH 7.4 is presented. * indicates p ≤ 0.05.
